# Comparable Early Functional Recovery Between Robotics-Assisted Superior Transverse Atraumatic Reconstruction (STAR) Total Hip Arthroplasty and the Direct Anterior Approach: A Six-Month Propensity-Matched Study

**DOI:** 10.3390/jcm15124713

**Published:** 2026-06-17

**Authors:** Hong Yu Jared Chua, Adam Farid Ming Yang Tang, Jiawei Chen, Hee Nee Pang, Darren Keng-Jin Tay, Ming Han Lincoln Liow

**Affiliations:** Department of Orthopaedics, Singapore General Hospital, Singapore Health Services, Singapore 169608, Singapore

**Keywords:** robotic hip surgery, robotics-assisted hip arthroplasty, superior transverse atraumatic reconstruction, functional outcomes, patient satisfaction, minimal clinically important difference

## Abstract

**Purpose:** There is limited literature on the functional outcomes of robotics-assisted (RA) superior transverse atraumatic reconstruction approach (STAR) in total hip arthroplasty (THA). This study compares the early functional outcomes, patient satisfaction, expectation fulfilment and complication rates between STAR RA-THA and direct anterior approach (DAA) RA-THA. **Methods:** We retrospectively analyzed all primary RA-THA performed between January 2022 and December 2024 at a high-volume tertiary centre. Patients undergoing either the DAA or STAR THA with available 6-month outcomes were included. There was a total of 123 patients (74 DAA and 49 STAR). Propensity score matching was used to account for baseline differences, generating 49 matched pairs based on age, sex, body mass index (BMI), and American Society of Anesthesiologists (ASA) grade. Postoperative patient-reported outcome measures (PROMs) and the proportion achieving minimum clinically important difference (MCID) were compared between groups. PROMs included were the Oxford Hip Score (OHS), Western Ontario and McMaster University Arthritic Index (WOMAC), Short-Form 36 Health Survey (SF-36) subscale, patient satisfaction score and expectation fulfilment score. **Results:** The DAA group demonstrated significantly better postoperative day one (POD1) ambulation distance (*p* = 0.00390). No differences in PROMs, MCID, patient satisfaction and expectation fulfilment scores were noted at last follow-up. The RA-DAA group had a higher incidence of meralgia paresthetica (*p* = 0.00260). **Conclusions:** The STAR RA-THA demonstrated early functional recovery comparable to DAA RA-THA. Although POD1 ambulation distance was greater in the DAA group, at six months, both approaches had similar functional outcomes, patient satisfaction and low complication rates.

## 1. Introduction

The field of total hip arthroplasty (THA) continually innovates, aiming to improve surgical outcomes and patient satisfaction [1]. In recent times, the utilization of direct anterior approach (DAA) THA as the standard choice has increased. Large registry data from countries like the United States and Netherlands show more than 28 and 40% increases in utilization of DAA THA from 2007 to 2020 respectively [2,3]. However, the utilization of DAA THA comes with its own set of challenges. Given its relatively steep learning curve, studies have shown an increase in rates of revision and complications in the first 100 cases of DAA THA performed by a surgeon [4,5].

More recently, a refinement of the traditional posterolateral approach (PLA) known as the superior transverse atraumatic reconstruction (STAR) THA has emerged as an excellent alternative. The STAR technique preserves the key advantages of the PLA, enabling an extensile approach with excellent visualization of the hip joint for accurate component positioning. By maintaining piriformis integrity and minimizing soft tissue disruption, the STAR technique has been associated with lower intraoperative complication rates [3,6,7,8], reduced postoperative dislocation risk, and improved hip stability [6].

A recent study compared mid-term outcomes of the DAA and STAR THA for primary hip osteoarthritis patients [9], demonstrating that both approaches achieved excellent 2-year patient-reported outcome measures (PROMs) and high rates of minimum clinically important difference (MCID) attainment. However, the technologically assisted DAA THA cohort demonstrated significantly higher functional scores, likely attributable to better component positioning, offset optimization, and limb-length restoration achieved with intraoperative fluoroscopy or robotic guidance. Notably, despite these differences, the manual STAR approach THA produced comparable satisfaction and MCID attainment rates at 2 years. Importantly, differences attributable to surgical approach are most evident in the early postoperative period. Hence, this prompted us to investigate the early postoperative benefit of the two surgical approaches in the context of robotic assistance [10,11,12].

To the best of our knowledge, no studies have compared the early outcomes of robotics-assisted (RA) DAA and STAR THA. Therefore, the primary endpoint of this study is to compare the early postoperative outcomes of DAA and STAR RA-THA, examining (1) postoperative day 1 (POD1) outcomes, (2) PROMs, patient satisfaction and (3) complications at the 6-month postoperative mark.

## 2. Materials and Methods

**Figure 1:**
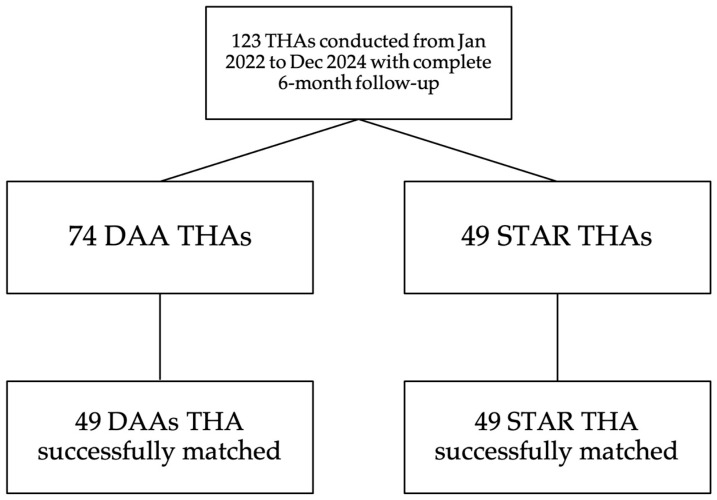
Study cohort derivation and matching process. THA = total hip arthroplasty; DAA = direct anterior approach; STAR = superior transverse atraumatic reconstruction.

This study received ethical approval from our institution (ECOS 2024–4046) and utilized deidentified patient data. We retrospectively reviewed all primary RA-THA performed at a high-volume surgical centre between January 2022 and December 2024. Procedures were performed by two fellowship-trained arthroplasty surgeons, each consistently utilizing a single surgical approach (DAA or STAR). Both surgeons were experienced in the two approaches. Both surgeons employed robotic assistance with the Mako robotic arm-assisted total hip™ (Stryker Corporation, Kalamazoo, MI, USA). Indications for surgery included primary and secondary hip osteoarthritis. Patients were not blinded to the choice of surgical approach. Inclusion in this study required availability of complete 6-month postoperative outcome data. A total of 123 cases met inclusion criteria, comprising 74 DAA cases and 49 STAR THA cases (Figure 1).

### 2.1. Surgical Technique

#### 2.1.1. DAA THA

The DAA THA was performed with the patient in the supine position on a traction table. The approach to the hip capsule was through the internervous and intermuscular interval between the tensor fascia latae and sartorius muscle. Following capsular exposure and napkin-ring femoral neck osteotomy, acetabular and femoral preparation were guided by the MAKO^®^ robotic arm-assisted system. Intraoperative mapping was registered to match with preoperative computed tomographic (CT) imaging to generate a three-dimensional (3D) reconstruction, enabling safe haptic-guided reaming and implant positioning. Final reduction was performed without capsular repair, followed by standard wound closure.

#### 2.1.2. STAR THA

The STAR approach was performed as previously described by Kenanidis et al. [7]. A posterior-based exposure involving splitting of the gluteus maximus fibres was utilized followed by identification and preservation of the piriformis muscle. The short external rotators were tenotomized and the capsule was inserted via an “inverted J” incision. After, robotics-assisted preparation of the acetabulum and femur was conducted using the MAKO^®^ robotic arm-assisted system. Intraoperative mapping was registered to match with preoperative computed tomographic (CT) imaging to generate a three-dimensional (3D) reconstruction, enabling safe haptic-guided reaming and implant positioning. Final reduction was performed without capsular repair, followed by standard wound closure.

#### 2.1.3. Standardized Postoperative Protocol

All patients were managed according to a standardized institutional postoperative protocol. This included same-day mobilization with physiotherapy initiated on POD0, routine implementation of an Enhanced Recovery After Surgery (ERAS) pathway with an expected 23 h length of stay, and a multimodal, opioid-free postoperative analgesic regimen. Analgesia comprised paracetamol and non-steroidal anti-inflammatory drugs (NSAIDs) in the absence of contraindications. The protocol was consistently applied across all surgical approaches and study participants.

### 2.2. Clinical Evaluation

Clinical assessments were conducted preoperatively, 24 h postoperatively, and at six months by experienced physiotherapists. On POD1, pain score, ambulation distance and hip flexion ROM were assessed. At the 6-month mark, PROMs included the Oxford Hip Score (OHS) [13], Western Ontario and McMaster University Arthritic Index (WOMAC) [14], and Short-Form 36 Health Survey (SF-36) scores [15], as well as patient satisfaction and expectation fulfilment scores. The OHS was graded out of 60, with a lower score indicating better outcomes. For patient satisfaction, a score between 1 and 3 was deemed satisfied, and scores between 4 and 6 were considered dissatisfied. For expectation fulfilment, scores of 1–3 meant that expectations were fulfilled and 4–6 meant that expectations were not met. Proportion of minimal clinically important difference attainment (MCID) for the SF-36 subscale scores and OHS were also studied to determine the effectiveness of the interventions. MCID thresholds were extracted from the current literature, with specific benchmarks set at 5 points for OHS [16]; 29.26, 26.54, and 25.91 for WOMAC pain, stiffness and physical function, respectively; 20.40, 10.78, 14.67, 0.40, 10.14, 25.97, 6.45, and 8.99 for SF-36 physical function, role physical, bodily pain, general health, vitality, social function, role emotional and mental health, respectively [17].

### 2.3. Statistical Analysis

Data analyses were performed using RStudio (Version 2026.04.0, Posit PBC, USA) and Microsoft Excel (Microsoft, Washington, USA). A priori power analysis (two-tailed alpha = 0.05; minimum power = 0.80; effect size = 0.6) indicated that 45 participants per group would be required to detect a statistically significant difference. Propensity score matching was employed to reduce baseline confounding between the THA groups. Scores were derived using logistic regression and patients were matched in a 1:1 ratio using a greedy algorithm with a calliper of 0.2 standard deviations [18]. Matching variables were age, sex, body mass index (BMI), and American Society of Anaesthesiologist (ASA) grade. A total of 49 matched pairs were generated.

Statistical significance was defined at *p* ≤ 0.05. Continuous variables were presented as the mean and standard deviation of the mean. Student’s unpaired *t*-tests compared quantitative variables between the two groups. Two-proportion *Z*-test was utilized to compare patient satisfaction and fulfilment of expectation.

Data distribution was assessed prior to parametric testing using visual inspection and normality testing. Given the relatively small sample size, findings should therefore be interpreted cautiously with consideration of potential type I error.

## 3. Results

### 3.1. Patient Demographic

There was no statistically significant difference between the two groups regarding age, sex, BMI and ASA grade after matching (Table 1).

### 3.2. Clinical Outcomes

On postoperative day 1 (POD1), there was no difference in postoperative pain, hip flexion or length of stay (LOS) (*p* > 0.05). The DAA THA patients had a greater ambulation distance than the STAR THA patients (29.5 ± 22.4 vs. 19.0 ± 9.87 m, *p* < 0.05) (Table 2).

At the 6-month postoperative mark, DAA and STAR THA demonstrated no significant differences across the PROMs assessed (Table 3). The DAA THA group fared better in OHS, WOMAC physical function, SF-36 physical function, general health and social function. On the other hand, the STAR THA group fared better in WOMAC pain, stiffness, SF-36 role physical, bodily pain, vitality, role emotional and mental health. These differences, however, were insignificant. The proportion of patients achieving MCID in both groups was similar (Table 4).

There was no significant difference in the proportion of patients reporting satisfaction or expectation fulfilment between the DAA and STAR groups (Table 5).

A total of twelve complications were noted in this study. There was one substantial complication of hip dislocation in the DAA THA group that did not require reoperation, nine cases of meralgia paresthetica (MP) and two complications of deep vein thrombosis in the STAR THA group that were treated medically (Table 6). Notably, the incidence of meralgia paresthetica (MP) was higher in the DAA THA group (Table 7).

## 4. Discussion

Surgical approach may influence early recovery following THA, when differences in soft tissue preservation are clinically most apparent. This study evaluated early outcomes of STAR RA-THA compared with DAA. At six months, both approaches demonstrated comparable pain relief, functional recovery, and complication profiles.

### 4.1. POD1 Outcomes

No statistically significant difference in POD1 pain scores was observed between groups, although scores trended lower in the STAR THA group. This observation parallels the findings of Alessio-Mazolla et al. who reported that patients undergoing posterior-based approaches required less postoperative analgesia than those undergoing anterior approaches [19]. This contrasts with findings from Wang et al. [20], Bovonratwet et al. [21] and Miller et al. [22] who reported significantly lower POD1 pain in DAA THA relative to posterior approaches. The uniformly low pain scores (mean 1.2 out of 10) observed in both groups likely reflects the soft tissue-sparing characteristics of the DAA and STAR techniques. While DAA patients achieved slightly greater POD1 ambulation distances, consistent with its early-mobilization advantage [20,21,22], no differences were observed in pain, ROM, flexion, or length of stay. Overall, both STAR and DAA THA demonstrated comparable early postoperative outcomes in terms of pain control and functional recovery.

Although the DAA cohort demonstrated significantly greater POD1 ambulation distance, the clinical significance of an approximately 10 m difference remains uncertain. It is unclear whether such a difference translates into meaningful long-term functional benefit.

### 4.2. Six-Month Postoperative Outcomes

Many studies demonstrated superior functional outcomes of DAA THA compared to PLA THA up to the 1-year postoperative mark [23,24]. In comparison, our study found no difference in functional outcomes of DAA and STAR THA at the 6-month postoperative mark. Several factors may account for this discrepancy. Firstly, the avoidance of muscle splitting and minimization of soft tissue disruption in both approaches plays a pivotal role in enhancing early functional outcomes [25,26]. Notably, the STAR and DAA techniques preserve the piriformis muscle vis-à-vis the traditional PLA which sacrifices it. Piriformis preservation, as described by Minokawa et al. [27], was found to have superior functional outcomes when compared to patients who underwent piriformis reattachment in THA. Additionally, standardized and robust postoperative physiotherapy protocols in our institution contribute to the excellent early functional outcomes in both cohorts, consistent with findings of Masaracchio et al. [28] and Chojnowska et al. [29].

Augmentation with RA may further reduce approach-related variability by enhancing implant positioning, restoring native hip anatomy, and reducing outliers in cup orientation and leg-length discrepancy [30,31]. Several recent studies, such as those by Konishi et al. [32] and Hayashi et al. [33], reported improved radiographic accuracy with RA-THA when compared to manual THA. Beyond radiographic advantages, RA-THA may also confer improved functional outcomes and PROMs as described by Ng et al. [34] and Bukowski et al. [35]. This is further supported by Schneider et al. [36] who reported that replicating the native hip centre of rotation and optimizing femoral offset is associated with improved functional benefit. However, while RA-THA has been studied via posterior and direct anterior approaches, the application of RA in STAR has not yet been evaluated, highlighting an area that has not yet been well studied and underscoring the relevance of the present investigation.

### 4.3. Complication Rates

The rate of major complications in our study was minimal. One case of hip dislocation was observed in the DAA group, attributed to stem subsidence and loss of soft tissue tension, and did not require revision surgery. No substantial complications were observed in the STAR THA group. These excellent outcomes may be partly attributed to surgeon experience [37,38]. Additionally, the inherent anatomical advantages of both approaches confer a lower risk of complications such as hip dislocation [39,40], with piriformis preservation in STAR THA proposed as one potential contributing factor. This aligns with prior studies describing low dislocation rates following STAR THA [7].

Importantly, RA may have further contributed to the low dislocation rates observed. Anderson et al. [41] reported that RA-THA improves component positioning and reduces dislocation risk compared to manual techniques. Both surgeons in our study employ preoperative spinopelvic screening and utilize robotic software (MAKO^®^ version 4) impingement modelling to fine-tune component placement to reduce dislocation risks.

The incidence of postoperative MP was significant in DAA THA. This, however, did not affect patient satisfaction rates in the DAA RA-THA group. The lateral femoral cutaneous nerve (LCFN) is particularly vulnerable due to its variable course along the anterior superior iliac spine and the typical incision of DAA THA [42,43]. While MP is usually self-limiting and does not typically have a clinically meaningful impact [44,45,46] on outcomes of THA, a small proportion of patients experience symptoms like severe pain and significant sensory disturbances requiring further intervention [47,48]. Hence, several strategies such as precise incision planning, gentle tissue handling and careful retractor placement should be considered to reduce the risk of MP [49,50].

### 4.4. Translation to Long-Term Outcomes

While the present study demonstrated differences in early postoperative recovery outcomes, it remains unclear whether these short-term advantages translate into sustained long-term functional benefits, improved implant survivorship, or reduced complication rates. As such, longer-term follow-up studies are required to determine the clinical significance and durability of these early findings.

### 4.5. Stem Type and Outcomes

In our study, the DePuy Synthes ACTIS^®^ cementless femoral stem and Stryker Insignia^®^ hip stems were used for all the cases. Both stems are triple-tapered femoral components designed to achieve metaphyseal fixation while preserving proximal femoral bone stock. This standardization reduces implant-related confounding and allows a more isolated comparison of surgical approach-related outcomes. Future studies comparing different femoral stem designs may help determine whether stem type influences PROMs [51].

### 4.6. Strengths

The strengths of our study include the following: (i) propensity score matching to reduce confounders, (ii) incorporation of multiple validated hip PROMs, and (iii) the completion of all surgical procedures by fellowship-trained surgeons which minimizes the learning curve of both approaches. The confounding effect of individual skill is also mitigated by the strict adherence to identical perioperative care pathways; (iv) surgeries were performed at a single institution with standardized postoperative protocols, (v) and this is one of few studies comparing POD1 outcomes, early functional outcomes, complication rates and patient satisfaction of the STAR THA with DAA THA.

### 4.7. Limitations

This study is not without limitations, which include the following: (i) There is likely multifactorial influence on functional outcomes such as radiographic parameters of the STAR and DAA THA, although its influence is likely limited as both groups were performed with robotic assistance. The authors plan to extend this work with detailed radiographic assessments and correlate them with postoperative functional outcomes in future analyses. (ii) The retrospective design of this study introduces potential selection bias. However, this was addressed through propensity score matching to adjust for confounding variables, thereby improving the validity of intergroup comparisons. (iii) All surgeries were performed in a single institution by highly experienced fellowship-trained surgeons, and the small sample size limits generalizability and reproducibility to other surgeons and institutions. (iv) Propensity analysis does not account for all potential confounding factors such as smoking and other comorbidities which are known to affect outcomes after THA. (v) Patients were not blinded to the surgical approach used. Given that DAA THA is often associated with superior functional outcomes due to marketing forces, the absence of blinding may have introduced expectation-related bias. However, this was not reflected in our findings. (vi) The 6-month follow-up precludes analysis of long-term survivorship. However, this timeframe was selected specifically to isolate early postoperative benefits of the surgical approach, which are most pronounced in the early recovery phase. (vii) Finally, each surgeon in this study utilized a single surgical approach. As a result, surgeon-specific factors including surgical philosophy, technical execution, perioperative decision-making and rehabilitation emphasis may influence outcomes independently of surgical approach. Therefore, effects of surgeon experience and surgical approach cannot be completely separated in the present study.

## 5. Conclusions

The STAR RA-THA demonstrated early functional recovery comparable to DAA RA-THA. Notably, the DAA RA-THA group exhibited greater ambulation distance at POD1 but similar POD1 pain scores. However, at six months, both approaches had similar functional outcomes and patient satisfaction and low complication rates. Longer-term follow-up is required to evaluate whether these findings translate to sustained functional benefits.

## Figures and Tables

**Table 1:** Group demographics matched. BMI = Body Mass Index; ASA = American Society of Anaesthesiologist.

	DAA	STAR	Significance/*p*-Value
Total no. of hips (patients)	49	49	
Gender			
Male	13	15	n.s
Female	36	34	
BMI/kg m^−2^	26.3 ± 4.19	26.4 ± 4.04	n.s
Age/years	69.3 ± 8.05	69.2 ± 8.01	n.s
ASA grade	2.00 ± 0.204	2.00 ± 0.354	n.s

**Table 2:** Immediate postoperative outcomes. ROM = range of motion.

	DAA	STAR	Significance/*p*-Value
Pain score (out of 10)	1.19 ± 1.27	1.15 ± 1.34	n.s
Flexion ROM (°)	92.1 ± 25.1	86.7 ± 14.6	n.s
Ambulation distance (m)	29.5 ± 22.4	19.0 ± 9.87	0.00390
Length of stay	1.57 ± 1.20	2.45 ± 3.20	n.s

**Table 3:** Comparison of pre and 6-month postoperative outcomes of DAA vs. STAR THA.

	DAA	STAR	Significance/*p*-Value
Oxford Hip Score			
Pre-op	38.0 ± 7.99	38.7 ± 9.38	n.s
6 months	16.4 ± 5.16	16.9 ± 5.78	n.s
WOMAC Pain			
Pre-op	53.6 ± 19.7	54.7 ± 24.8	n.s
6 months	96.7 ± 9.81	98.4 ± 4.79	n.s
WOMAC Stiffness			
Pre-op	58.5 ± 26.8	64.4 ± 33.4	n.s
6 months	91.0 ± 16.5	94.3 ± 14.3	n.s
WOMAC Physical Function			
Pre-op	48.6 ± 18.4	47.1 ± 23.5	n.s
6 months	90.3 ± 6.72	86.2 ± 14.2	n.s
SF-36 Physical Function			
Pre-op	30.1 ± 25.3	27.7 ± 26.5	n.s
6 months	68.3 ± 23.8	68.0 ± 25.1	n.s
SF-36 Role Physical			
Pre-op	8.33 ± 23.3	12.8 ± 31.1	n.s
6 months	56.3 ± 45.0	57.9 ± 44.3	n.s
SF-36 Bodily Pain			
Pre-op	29.0 ± 17.0	31.4 ± 25.7	n.s
6 months	61.6 ± 23.9	72.1 ± 29.7	n.s
SF-36 General Health			
Pre-op	63.5 ± 19.6	66.3 ± 26.5	n.s
6 months	74.8 ± 17.5	72.8 ± 24.6	n.s
SF-36 Vitality			
Pre-op	63.3 ± 19.7	63.5 ± 24.6	n.s
6 months	78.5 ± 15.2	79.5 ± 17.7	n.s
SF-36 Social Function			
Pre-op	50.5 ± 37.7	42.9 ± 35.8	n.s
6 months	89.6 ± 18.3	87.5 ± 24.5	n.s
SF-36 Role Emotional			
Pre-op	74.8 ± 41.7	77.6 ± 41.0	n.s
6 months	91.2 ± 24.6	93.0 ± 24.7	n.s
SF-36 Mental Health			
Pre-op	73.0 ± 20.9	75.2 ± 20.7	n.s
6 months	84.7 ± 10.0	86.8 ± 12.8	n.s

**Table 4:** Comparison of proportion of patients achieving MCID at 6 months.

	DAA (%)	STAR (%)	Significance/*p*-Value
Oxford Hip Score (5)	100	100	n.s
Western Ontario McMaster University Osteoarthritis Index			
Pain (29.26)	77.6	73.5	n.s
Stiffness (26.54)	63.3	49.0	n.s
Physical Function (25.91)	87.8	75.5	n.s
Short-form 36			
Physical Function (20.40)	85.7	77.6	n.s
Role Physical (10.78)	81.6	71.4	n.s
Bodily Pain (14.67)	91.8	87.8	n.s
General Health (0.40)	83.7	73.5	n.s
Vitality (10.14)	61.2	63.3	n.s
Social Function (25.97)	57.1	61.2	n.s
Role Emotional (6.45)	18.4	20.4	n.s
Mental Health (8.99)	59.2	57.1	n.s

Recommended MCID values are shown in parenthesis.

**Table 5:** Satisfaction and fulfilment of expectations at 6 months.

	DAA (*n* = 49)	STAR (*n* = 49)	Significance/*p*-Value
Satisfied (%)	100 (49/49)	98.0 (48/49)	n.s
Dissatisfied (%)	0 (0/49)	2.00 (1/49)	
Expectations fulfilled (%)	100 (49/49)	98.0 (48/49)	n.s
Expectations not fulfilled (%)	0 (0/49)	2.00 (1/49)	

**Table 6:** Postoperative complications.

Patient No.	DAA	Reoperation/Revision Surgery Required?
1	Hip Dislocation	No
2	Meralgia Paresthetica	No
3	Meralgia Paresthetica	No
4	Meralgia Paresthetica	No
5	Meralgia Paresthetica	No
6	Meralgia Paresthetica	No
7	Meralgia Paresthetica	No
8	Meralgia Paresthetica	No
9	Meralgia Paresthetica	No
10	Meralgia Paresthetica	No
Patient No.	STAR	Reoperation/Revision Surgery Required?
11	Deep Vein Thrombosis	No
12	Deep Vein Thrombosis	No

**Table 7:** Incidence of meralgia paresthetica.

	DAA (*n* = 49)	STAR (*n* = 49)	Significance/*p*-Value
Present (%)	18.4 (9/49)	0 (0/49)	0.00260
Absent (%)	81.6 (40/49)	100 (49/49)	

## Data Availability

The data supporting the findings of this study contain sensitive patient information and cannot be shared publicly due to ethical and privacy considerations. Access to anonymized data may be granted by the corresponding author upon reasonable request and following approval by the relevant institutional review board.
